# Supplementary material for: mRNA-Loaded Lipid Nanoparticles Targeting Dendritic Cells for Cancer Immunotherapy
Source: Pharmaceutics. 2022 Jul 28;14(8):1572. doi: 10.3390/pharmaceutics14081572 (PMC9413374; doi:10.3390/pharmaceutics14081572)
Supplement: Supplementary file 1 [file pharmaceutics-14-01572-s001.zip › pharmaceutics-1832994-supplementary.pdf]

SUPPORTING INFORMATION

mRNA-loaded lipid nanoparticles targeting dendritic cells for cancer immunotherapy

Kosuke Sasaki†, Yusuke Sato†, Kento Okuda, Kazuki Iwakawa, Hideyoshi Harashima**

Laboratory for Molecular Design of Pharmaceutics, Faculty of Pharmaceutical Sciences, Hokkaido University, Kita-12, Nishi-6, Kita-Ku, Sapporo 060-0812, Japan

† These authors contributed equally to this work.

**Corresponding authors:*
Yusuke Sato E-mail address: y_sato@pharm.hokudai.ac.jp Tel: +81 11 706 3734 Fax: +81 11 706 3734
Hideyoshi Harashima E-mail address: harasima@pharm.hokudai.ac.jp Tel: +81 11 706 3919 Fax: +81 11 706 3734

Supplementary methods: synthesis of CL7H6

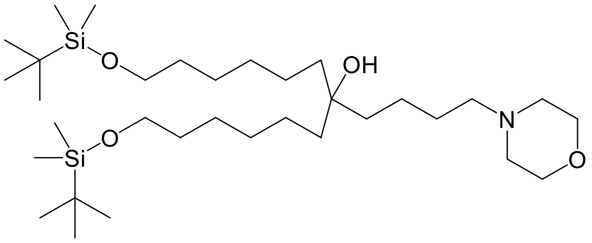

Synthesis of 2,2,3,3,19,19,20,20-octamethyl-11-(4-morpholinobutyl)-4,18-dioxa-3,19-disilahenicosan-11-ol. Morpholine (10.5 mL, 120 mmol) and DCM (29 mL) were added to 11-((*tert*-butyldimethylsilyl)oxy)-5-(6-((tert-butyldimethylsilyl)oxy)hexyl)-5-hydroxyundecyl 4-methylbenzenesulfonate (13.7 g, 20 mmol) and the reaction mixture was stirred at room temperature for 5 days. After evaporation of the solvent, the residue was suspended in EtOAc and filtered. The filtrate was washed with water (2×50 mL), and then brine (50 mL). The organic phase was dried over Na_2SO_4. Evaporation of the solvent gave the crude product as a pale yellow oily residue. The residue was purified by flash chromatography (SiO_2, DCM/MeOH). This gave 10.3 g (86%) of compound 2,2,3,3,19,19,20,20-octamethyl-11-(4-morpholinobutyl)-4,18-dioxa-3,19-disilahenicosan-11-ol as a pale yellow oil.

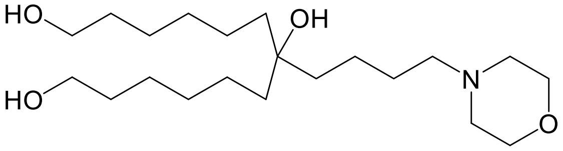

Synthesis of 7-(4-morpholinobutyl)tridecane-1,7,13-triol. 2,2,3,3,19,19,20,20-Octamethyl-11-(4-morpholinobutyl)-4,18-dioxa-3,19-disilahenicosan-11-ol (10.3 g, 17.1 mmol) was dissolved in MeOH (20 mL) and the solution was cooled on ice. After adding a 12 N HCl solution (5 mL) to the solution, the reaction mixture was stirred overnight at room temperature. After evaporation of the solvent, the residue was diluted with a NaOH solution (0.5 M, 100 mL) and then extracted with EtOAc (2×100 mL). The combined organic phases were dried over Na_2SO_4. Evaporation of the solvent gave crude as a pale yellow oily residue. The residue was purified by flash chromatography (SiO_2, DCM/MeOH). This gave 3.93 g (53%) of 7-(4-morpholinobutyl)tridecane-1,7,13-triol as a colorless oil.

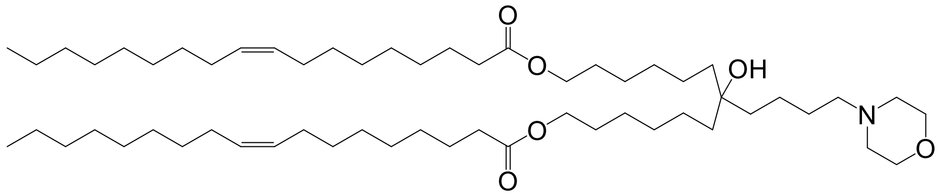

Synthesis of 7-hydroxy-7-(4-morpholinobutyl)tridecane-1,13-diyl dioleate (CL7H6). 7-(4-Morpholinobutyl)tridecane-1,7,13-triol (3.93 g, 10.5 mmol) was dissolved in anhydrous DCM (30 mL) and oleic acid (6.54 g, 23.2 mmol), DMAP (283 mg, 2.32 mmol) and EDCI-HCl (5.05 g, 26.3 mmol) were added to the mixture. The reaction mixture was stirred at ambient temperature for 8 hours. After evaporation of the reaction mixture, the residue was suspended with EtOAc (100 mL) and washed with, a NaOH solution (0.5 M, 100 mL) and then brine (50 mL). The organic phase was dried over Na_2SO_4. Evaporation of the solvent gave the crude product as a pale yellow oily residue. The residue was purified by flash chromatography (SiO_2, DCM/MeOH/NH_4OH). This gave 9.17 g (97%) of 7-hydroxy-7-(4-morpholinobutyl)tridecane-1,13-diyl dioleate (CL7H6) as a pale yellow oil.

Supplementary Figures

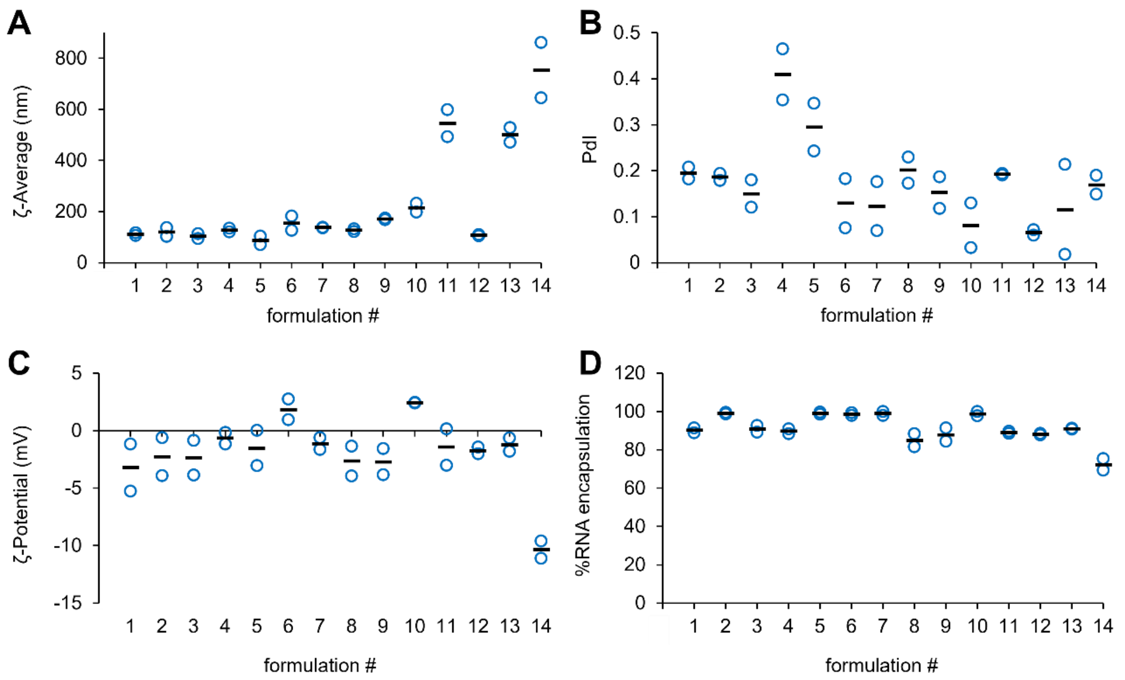

Figure S1. Reproducibility of the physicochemical properties of the 14 LNPs (A-1 to A-14) examined in screening A. ζ-Average (A), PdI (B), ζ-potential (C), and %RNA encapsulation (D) of all of the LNPs were reliably reproduced in two technically independent experiments.

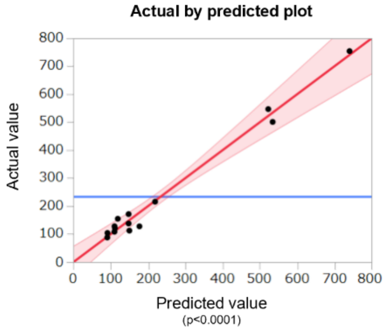

Summary of Fit	
R^2	0.985
Adjusted R^2	0.973
Root Mean Square Error	34.33
Mean	233.5
n	14

ANOVA Results			
Source	Degrees of Freedom	Sum of Squares	Mean Square
Model	6	555,086	92,514
Error	7	8,250	1,179
Total	13	563,336	

F-Test Results		
	F Ratio	Prob > F
ANOVA	78.50	< 0.0001

Parameter Estimates				
Term in Model	Estimate	Std. Error	t Ratio	Prob > |t|
Intercept	147.775	17.1654	8.61	< 0.0001
%DOPE%(10,40)	-93.43	10.85635	-8.61	< 0.0001
%PEG(0.5,1.5)	-64.45	10.85635	-5.94	0.0006
NaCl conc. (mM)(0,400)	157.27	10.85635	14.49	< 0.0001
%DOPE*NaCl conc. (mM)	-112.8133	12.53584	-9.00	< 0.0001
%PEG*NaCl conc. (mM)	-44.54667	12.53584	-3.55	0.0093
NaCl conc. (mM)*NaCl conc. (mM)	119.975	20.31038	5.91	0.0006

Effect Tests			
Effect	Sum of Squares	F Ratio	Prob > F
%DOPE(10,40)	87,291.65	74.0636	< 0.0001
%PEG(0.5,1.5)	41,538.03	35.2434	0.0006
NaCl conc. (mM)(0,400)	247,338.53	209.8572	< 0.0001
%DOPE*NaCl conc. (mM)	95,451.36	80.9868	< 0.0001
%PEG*NaCl conc. (mM)	14,883.04	12.6277	0.0093
NaCl conc. (mM)*NaCl conc. (mM)	41,125.72	34.8936	0.0006

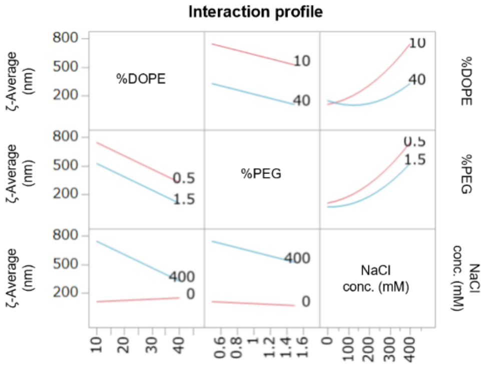

Figure S2. Statistical information for the effective design-based model selection for definitive screening design to predict the ζ-average of the LNPs (A-1 to A-14).

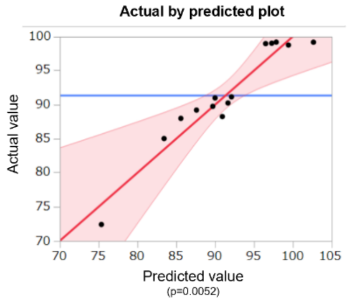

Summary of Fit	
R^2	0.925
Adjusted R^2	0.838
Root Mean Square Error	3.002
Mean	91.44
n	14

ANOVA Results			
Source	Degrees of Freedom	Sum of Squares	Mean Square
Model	7	668.5	95.50
Error	6	54.09	9.015
Total	13	722.6	
F-Test Results			
	F Ratio	Prob > F	
ANOVA	10.59	0.0052	

Parameter Estimates				
Term in Model	Estimate	Std. Error	t Ratio	Prob > |t|
Intercept	91.441429	0.802443	113.95	< 0.0001
%CL(40,60)	2.5714286	0.982788	2.62	0.0398
%DOPE(10,40)	3.3371667	0.97548	3.42	0.0141
CL[CL4H6]	3.9714881	0.845849	4.70	0.0033
PEG[PEG-DMG]	1.8756548	0.845849	2.22	0.0684
NaCl conc. (mM)(0,400)	-3.356167	0.97548	-3.44	0.0138
%CL*NaCl conc. (mM)	3.5483333	1.096345	3.24	0.0178
%DOPE*NaCl conc. (mM)	5.3583333	1.096345	4.89	0.0027

Effect Tests			
Effect	Sum of Squares	F Ratio	Prob > F
%CL(40,60)	61.71429	6.8459	0.0398
%DOPE(10,40)	105.50540	11.7036	0.0141
CL	198.73624	22.0456	0.0033
PEG	44.32782	4.9172	0.0684
NaCl conc. (mM)(0,400)	106.71020	11.8372	0.0138
%CL*NaCl conc. (mM)	94.43002	10.4750	0.0178
%DOPE*NaCl conc. (mM)	215.33802	23.8872	0.0027

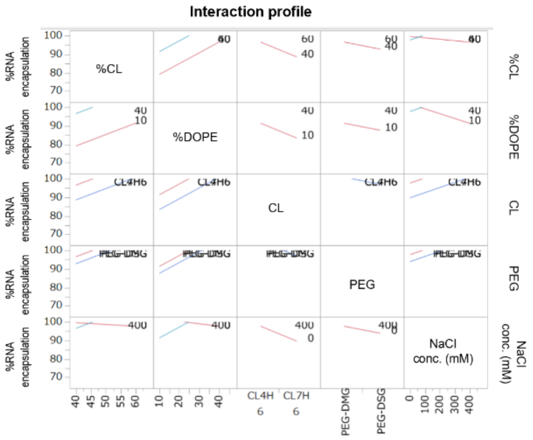

Figure S3. Statistical information for the effective design-based model selection for definitive screening design to predict the percentage of RNA encapsulation of the LNPs (A-1 to A-14).

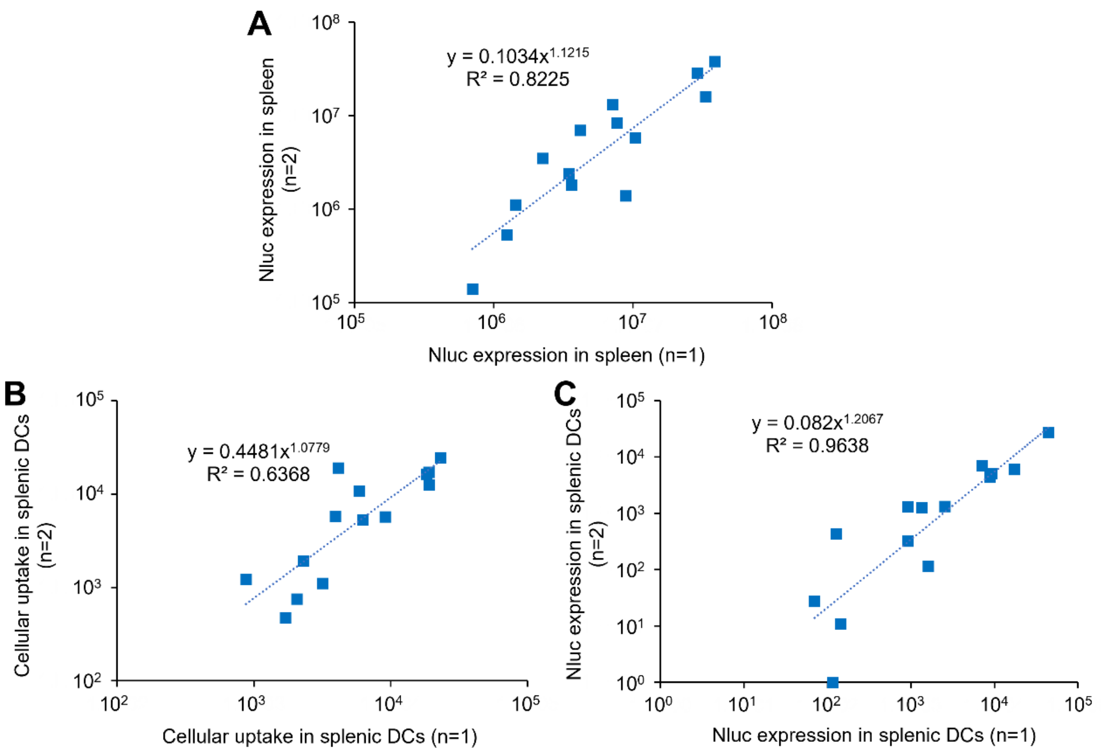

Figure S4. Reproducibility of outputs of the in vivo experiments in screening A. Nluc expression in whole spleen (A), cellular uptale by splenic DCs (B), and Nluc expression in splenic DCs (C) were reproduced between two technically independent experiments.

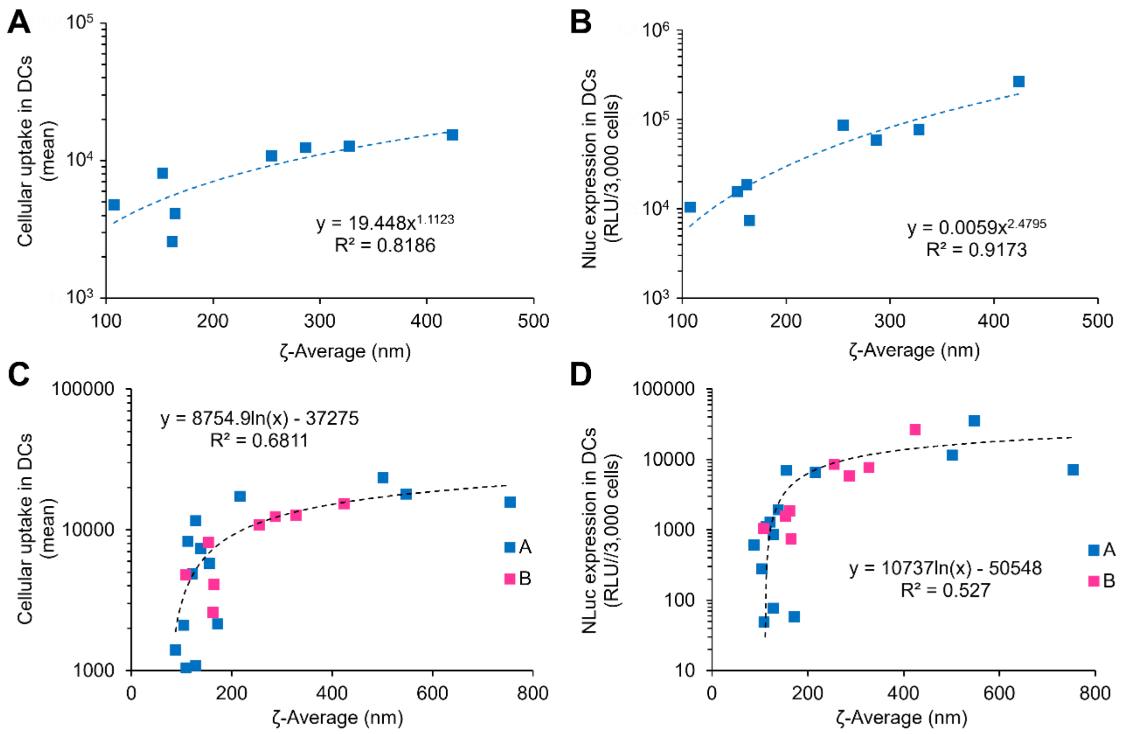

Figure S5. Correlation between ζ-average and cellular uptake or Nluc expression in splenic DCs. (A, B) Plots of cellular uptake (A) or Nluc expression (B) versus ζ-average of the LNPs examined in screening B (B-1 to B-8). (C, D) Overlaid plots of cellular uptake (C) or Nluc expression (D) versus ζ-average of the LNPs examined in both screening A and B.

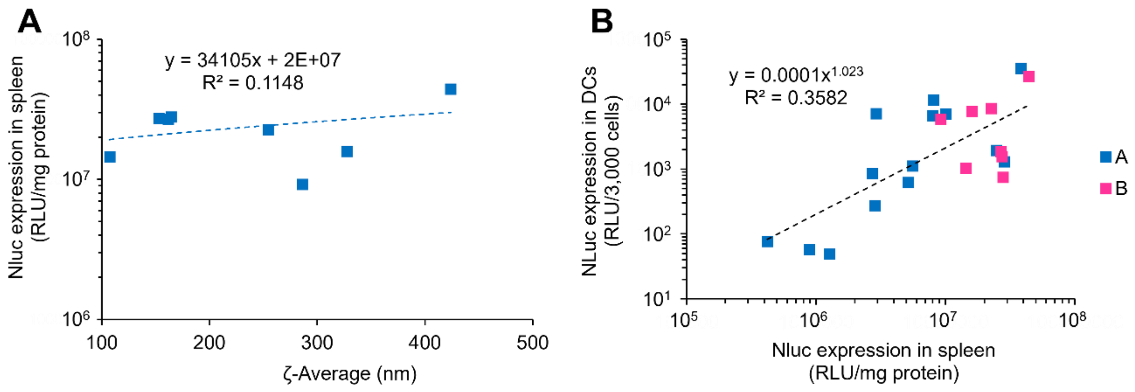

Figure S6. Correlation between ζ-average and Nluc expression. (A) Plots of Nluc expression in whole spleen versus ζ-average of the LNPs examined in screening B (B-1 to B-8). (B) Overlaid plots of Nluc expression in splenic DCs versus ζ-average of the LNPs examined in both screening A and B.

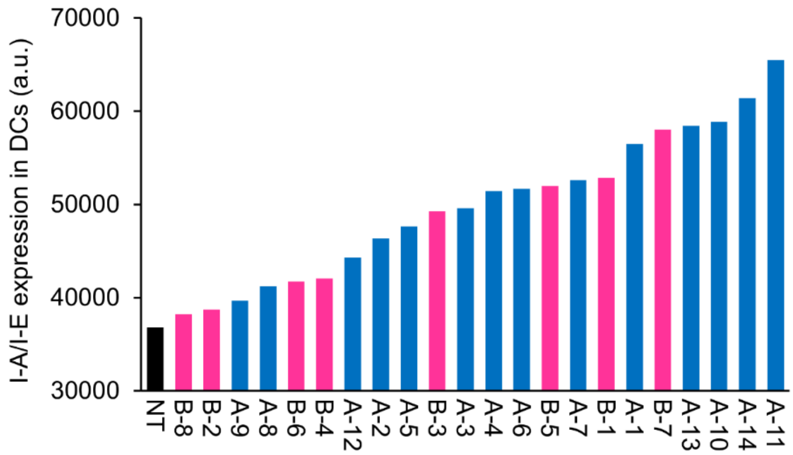

Figure S7. I-A/I-E expression in splenic DCs after intravenous injection of the LNPs examined in screening A and B.

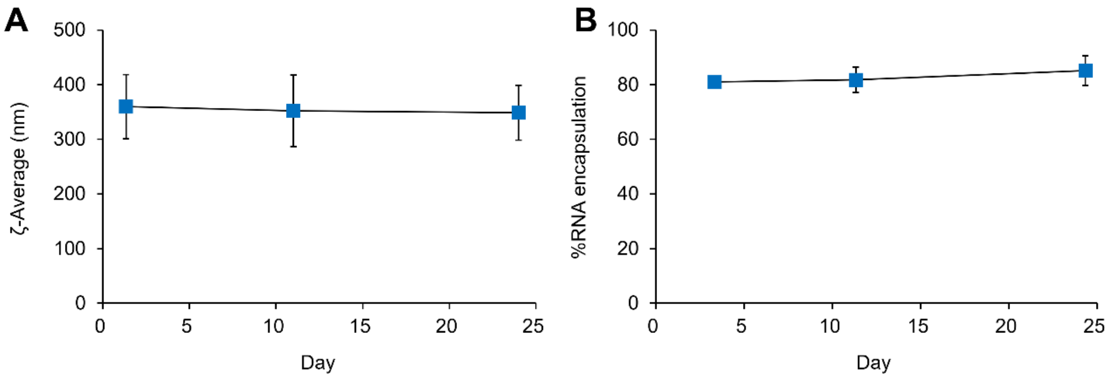

Figure S8. Stability of the A-11-LNPs. ζ-Average (A) and %RNA encapsulation (B) were measured at the indicated time points after storage at 4°C as a wet preparation. n=3.

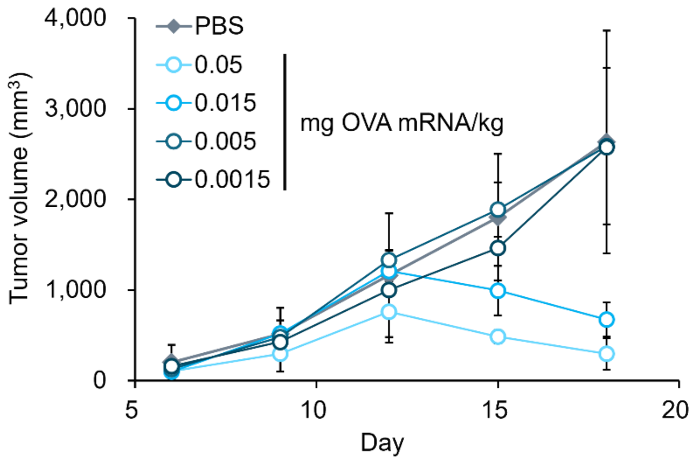

Figure S9. Examination of the dose of the OVA mRNA-loaded A-11-LNPs for therapeutic antitumor experiment. E.G7-OVA tumor bearing mice were intravenously injected with the A-11-LNPs on day 8 and 11 at the indicated doses. n=3.

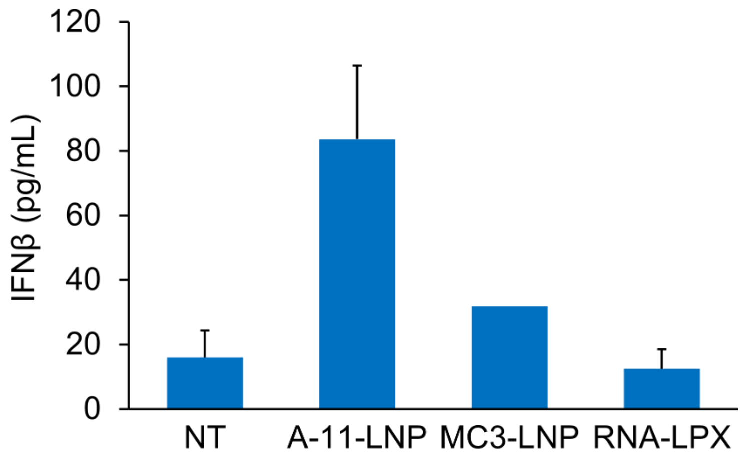

Figure S10. Serum IFNβ level after an intravenous injection of OVA mRNA-loaded formulations at a dose of 0.03 mg mRNA/kg. n=2-3.

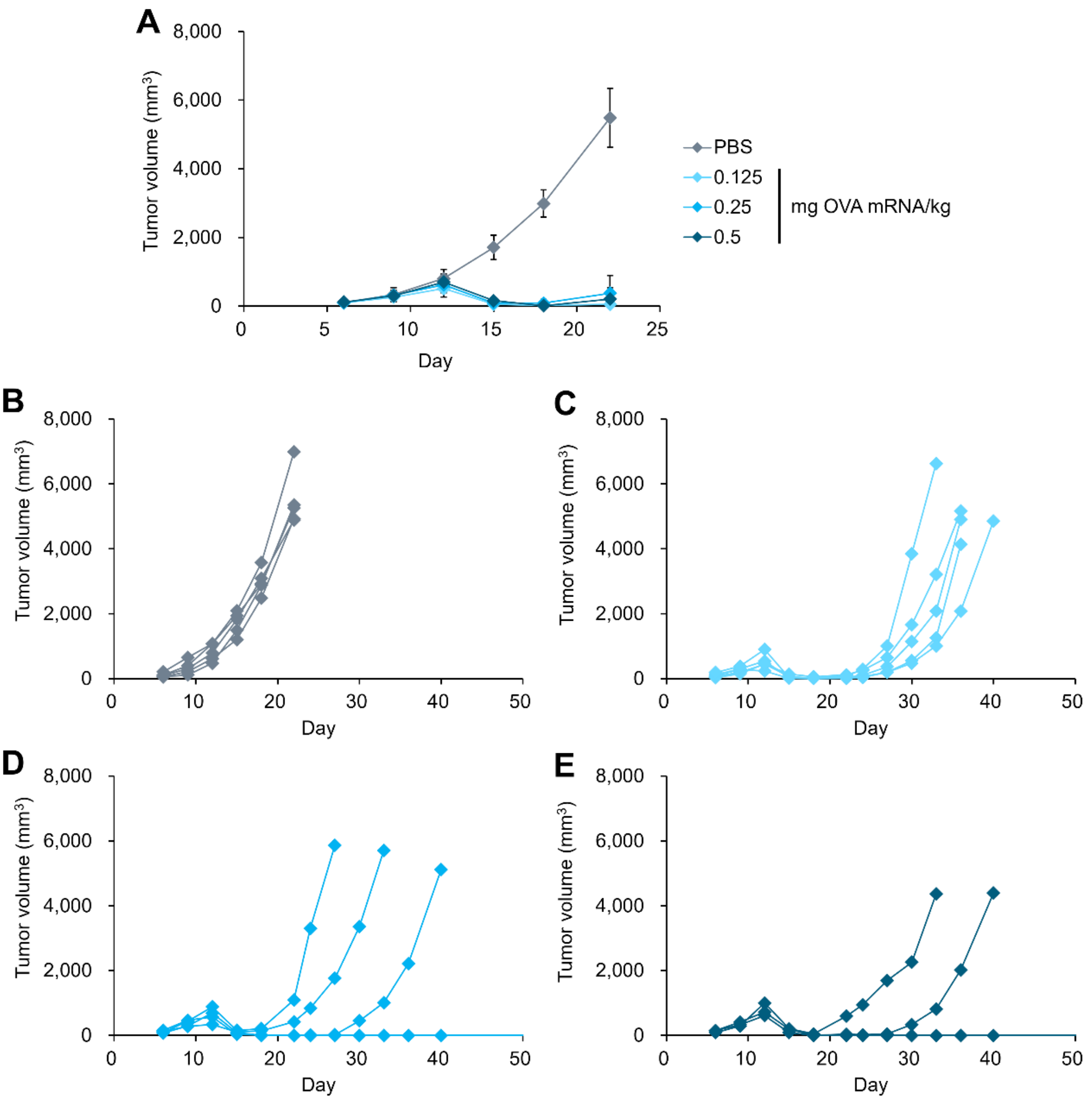

Figure S11. Therapeutic antitumor effect of the A-11-LNPs under high dose conditions. E.G7-OVA tumor bearing mice were intravenously injected with the OVA mRNA-loaded A-11-LNPs on day 7, 10, and 14 at the indicated doses. n=3-5. (A) Averaged tumor volumes in all groups. (B) Tumor volumes of individual mouse in PBS group (B) and in groups treated with 0.125 (C), 0.25 (D), and 0.5 (E) mg mRNA/kg.

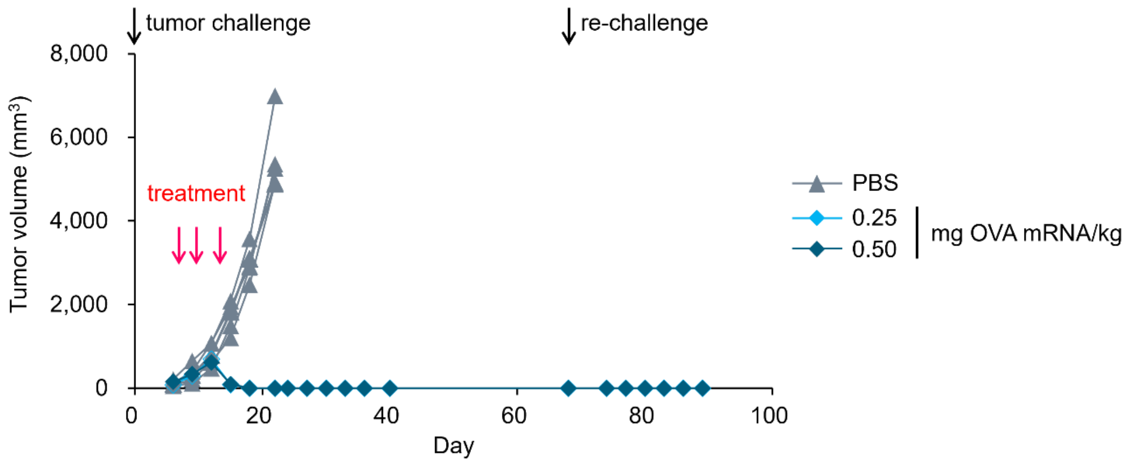

Figure S12. Examination of the formation of memory cells. E.G7-OVA tumor cells were re-challenged at 68 days after the initial tumor transplantation in two individuals who completely responded.

Supplementary tables

Table S1. Full data for the outputs of in vivo experiment in screening A

Entry	Cellular uptake (mean)			Nluc expression in splenic cells (RLU/3,000 cells)			Nluc expression in whole spleen (RLU/mg protein)
	B cells	Mφ	DCs	B cells	Mφ	DCs	
A-1	461.3	21825.0	8330.8	19.0	904.5	1115.0	5579921
A-2	289.5	11944.1	4854.5	66.5	3803.0	1299.5	28656815
A-3	189.1	4815.3	2108.3	1.0	847.0	279.0	2862791
A-4	459.2	30157.6	11608.3	-11.5	220.0	860.0	2718293
A-5	21.0	3557.0	1404.4	8.0	1052.0	617.5	5136667
A-6	131.7	19888.7	5769.6	58.5	3798.5	7072.5	10071588
A-7	422.0	17500.5	7382.4	43.5	5180.5	1941.5	24675672
A-8	133.0	1790.0	1087.9	24.0	22.5	77.0	420892
A-9	146.6	7116.6	2145.9	9.0	313.5	58.5	884859
A-10	974.4	56572.2	17332.4	67.0	3357.0	6614.5	8001441
A-11	462.2	65176.9	18026.2	71.5	9826.0	35818.5	38305663
A-12	52.2	10036.2	1048.8	-1.5	349.5	49.0	1272565
A-13	559.2	80380.1	23659.3	65.5	4960.5	11585.5	8091609
A-14	731.1	42212.5	15798.6	54.5	7006.5	7159.0	2914146

Table S2. Formulation conditions and physicochemical properties of the LNPs examined in screening B

Entry	%CL	%PL	%PEG	PEG	ζ-Average (nm)	PdI	ζ-Potential (mV)	%RNA encapsulation
B-1	50	10	0.75	PEG-DMG	327	0.14	-0.1	91.5
B-2	50	10	1.5	PEG-DSG	162	0.14	1.5	93.4
B-3	50	20	0.75	PEG-DSG	255	0.07	1.5	99.7
B-4	50	20	1.5	PEG-DMG	107	0.05	1.0	99.5
B-5	60	10	0.75	PEG-DSG	424	0.09	1.9	93.1
B-6	60	10	1.5	PEG-DMG	153	0.04	0.4	83.8
B-7	60	20	0.75	PEG-DMG	286	0.06	3.1	99.4
B-8	60	20	1.5	PEG-DSG	164	0.10	1.9	97.2

Table S3. Full data for the outputs of in vivo experiment in screening B

Entry	Cellular uptake (mean)			Nluc expression in splenic cells (RLU/3,000 cells)			Nluc expression in whole spleen (RLU/mg protein)
	B cells	Mφ	DCs	B cells	Mφ	DCs	
B-1	1486	77194	12792	132.8	193.2	7723.8	15975354
B-2	200	11174	2594	23.9	7271.0	1867.5	26838064
B-3	750	46550	10921	158.4	6885.7	8640.5	22656792
B-4	410	14011	4781	247.5	4324.0	1049.0	14425385
B-5	474	93818	15382	145.9	157.6	26794.5	44185259
B-6	584	32942	8146	28.1	11068.6	1563.5	27375609
B-7	788	54727	12465	85.8	1297.4	5881.5	9216984
B-8	253	18108	4121	149.3	6881.5	746.5	27828969
